# Perception of Binaural Cues Develops in Children Who Are Deaf through Bilateral Cochlear Implantation

**DOI:** 10.1371/journal.pone.0114841

**Published:** 2014-12-22

**Authors:** Karen A. Gordon, Michael R. Deighton, Parvaneh Abbasalipour, Blake C. Papsin

**Affiliations:** 1 Archie's Cochlear Implant Laboratory, The Hospital for Sick Children, Toronto, Ontario, Canada; 2 Department of Otolaryngology-Head and Neck Surgery, University of Toronto, Ontario, Canada; University of Montreal, Canada

## Abstract

There are significant challenges to restoring binaural hearing to children who have been deaf from an early age. The uncoordinated and poor temporal information available from cochlear implants distorts perception of interaural timing differences normally important for sound localization and listening in noise. Moreover, binaural development can be compromised by bilateral and unilateral auditory deprivation. Here, we studied perception of both interaural level and timing differences in 79 children/adolescents using bilateral cochlear implants and 16 peers with normal hearing. They were asked on which side of their head they heard unilaterally or bilaterally presented click- or electrical pulse- trains. Interaural level cues were identified by most participants including adolescents with long periods of unilateral cochlear implant use and little bilateral implant experience. Interaural timing cues were not detected by new bilateral adolescent users, consistent with previous evidence. Evidence of binaural timing detection was, for the first time, found in children who had much longer implant experience but it was marked by poorer than normal sensitivity and abnormally strong dependence on current level differences between implants. In addition, children with prior unilateral implant use showed a higher proportion of responses to their first implanted sides than children implanted simultaneously. These data indicate that there are functional repercussions of developing binaural hearing through bilateral cochlear implants, particularly when provided sequentially; nonetheless, children have an opportunity to use these devices to hear better in noise and gain spatial hearing.

## Introduction

Unlike sight, we are able to hear from all directions around us. This is important for survival and communication. Directional hearing helps us to listen to one voice amongst many by comparing sounds reaching our two ears. Sounds sources in space hit the outer ears at different places and angles producing unique and recognizable spectral patterns [1]. In addition, sounds away from mid-line reach the nearer ear more quickly and/or at a higher intensity than they do the other ear. The bilateral input is integrated in the bilateral auditory pathways into a fused image to answer what the sound is and the interaural cues are coded to indicate where it is coming from [1]. The importance of binaural hearing is highlighted by the significant implications for language development and educational outcomes when unilateral hearing loss occurs in childhood [2]–[4]. We have attempted to restore binaural hearing in children with bilateral deafness by providing cochlear implants in each ear [5] but face a number of challenges including the abnormal electrical input of the cochlear implant and the effects of deafness during early development.

The cochlear implant is surgically placed in each inner ear (cochlea) to bypass the impaired sensory system and electrically stimulate the auditory nerve on each side [6], [7]. The implants effectively provide access to sound and promote both auditory [8] and language development [9]. On the other hand, cochlear implants cannot restore normal pitch perception [10], [11] because the electrical evoked neural excitation patterns are abnormally wide spread and synchronous [12]–[14]. These issues also compromise access to binaural cues when cochlear implants are provided bilaterally. Although speech detection improves when children and adults use bilateral rather than unilateral implants [15]–[17], poorer than normal perception of binaural timing cues persist [18], [19], contributing to impaired localization of sound [20], [21].

Reduced sensitivity to interaural timing cues in adults occurs despite efforts to match place of stimulation on the two sides [22] and is markedly reduced when rates of electrical pulse presentation are increased [19]. The same problem occurs for normal hearing listeners for high frequency tonal stimuli because “cycle by cycle” processing of the fine structure of sounds is better preserved in low frequencies [23]. Binaural timing cues for high frequency sounds are carried by the amplitude changes in time, or envelope, of the signal which occur at slower rates [24]. Cochlear implant pulses do follow the envelope of the acoustic signal but the two devices operate independently, providing inconsistent binaural timing cues. Nonetheless, even when these issues are addressed, poor sensitivity to timing differences presented by electrical pulses remain [25]. This has been explained by the abnormally high synchronous inputs to the brainstem [26]. Proposed solutions have been to use low rates of electrical stimulation [19] and generate speech processing strategies which would highlight binaural timing differences [27]. The importance of matching pitch between the implants and optimizing binaural level and spectral cues has been emphasized for improving binaural hearing [28]–[30].

Given such challenges inherent to cochlear implants, can children who are deaf develop binaural hearing with these devices? Early findings suggest that children with bilateral implants have considerably poorer sound localization and perception of binaural cues than adult users who lost their hearing later in life [18], [31], [32]. Restoring binaural hearing to children is likely further complicated by the effects of deafness on the auditory pathways during important stages of auditory development.

Although it is clear that cochlear implantation should proceed as early as possible for oral speech and language development [6], [8], [33], cochlear implants were traditionally provided in only one ear. Many children with bilateral implants received one device and used it for some time before the other ear was implanted, promoting strengthening of the pathways from the stimulated ear with relatively immature responses persistent in pathways from the unstimulated side [34], [35]. Resulting asymmetries in auditory function were found in the auditory brainstem [36] and cortex [37], [38] and correlated with poorer speech perception in the newly implanted ear relative to the first [39], [40]. This was avoided in children bilaterally implanted within a 1.5 year span [36], [37].

Importantly, the asymmetric development promoted by unilateral implant use did not eliminate integration of binaural input in the brainstem [36] but severe impairments were found in perception of binaural cues. Initial results from our group and Litovsky and colleagues showed that children using bilateral implants, most with long inter-implant delays, could not detect even large changes in binaural timing differences [18], [32]. By contrast, they were able to detect changes in current level differences between devices [18], [32] and use envelope cues to better perceive a signal in noise than with unilateral implants [15], [16]. Given that binaural processing, at least, at the brainstem, was possible [36], we hypothesized that perception of binaural cues would be established in children receiving bilateral cochlear implants with long term use but that differences from normal would persist with increasing abnormalities for those children who had experienced longer durations of unilateral implant use.

## Materials and Methods

### Participants

The study protocol and consent procedure were approved by the Hospital for Sick Children's Research Ethics Board (#1000002954). A total of 95 adolescents/children consented to participate in this study. Written consent was obtained from caretakers or guardians on behalf of participants who were under 18 years of age at the time of enrollment and written assent was obtained in those children who were able to provide it. Three groups used bilateral cochlear implants and one group had normal hearing. The demographic details are shown in Table 1. The first group of bilateral cochlear implant users included 34 adolescents (12.64±3.49 years old at the first test) with long term unilateral implant use (implanted at 3.62±2.24 years and 9.02±2.80 years of unilateral use); they had been recently provided with a second cochlear implant. This group was tested at initial bilateral cochlear implant use (0.14±0.01 year), typically within the first month. Measures were repeated within the first year of bilateral implant use (0.86±0.03 year), typically ∼10 months of use, in 29 of these children. The second group (n = 16) also had several years of unilateral cochlear implant use (2.69±1.64 years of age at implant, 4.10±2.17 years of unilateral implant experience) at the time the second ear was implanted but the second surgery occurred at much younger ages than the first group (6.79±3.01 versus 12.64±3.49 years of age). This second group had used bilateral cochlear implants for many (5.30±1.55) years at the time of testing. The third group (n = 29) was implanted bilaterally in the same surgery at young ages (2.95±2.54 years) and also had long term bilateral implant experience at test time (4.31±1.10 years). Data from children with bilateral cochlear implants were compared with those from a group of 16 children and adolescents with normal hearing (9.16±2.19 years of age). Normal hearing was confirmed by ensuring that children could detect pure tones (250, 500, 1000, 2000, 4000, 8000 Hz) presented at 20 dB HL in each ear. Children responded to these stimuli by raising their hand when the sound was presented.

**Table 1:** Participant demographics.

	(n)	R:L 1^st^ CI	Age at first implant	Age at second implant	Inter-implant delay	Duration of hearing experience		Duration of bilateral hearing		Age at Test	
Adolescents with long inter-implant delays						Test 1	Test 2	Test1	Test2	Test 1	Test 2
	34	28∶6	3.62±2.24	12.64±3.49	9.02±2.80	9.16±2.81	9.92±2.97	0.14±0.01	0.86±0.03	12.79±3.51	13.82±3.63
Children with long inter-implant delays	16	13∶3	2.69±1.64	6.79±3.01	4.10±2.17	9.40±2.34		5.30±1.55		12.10±2.81	
Children with no inter-implant delays	29	n/a	2.95±2.54	2.95±2.54	0	4.31±1.10		4.31±1.10		7.49±2.12	
Children with normal hearing	16	n/a	n/a	n/a	n/a	9.16±2.19		9.16±2.19		9.16±2.19	

### Stimuli

The normal hearing group listened to 500 ms trains of clicks presented at 250 clicks/s and bilateral cochlear implant users listened to 500 ms trains of biphasic electrical pulses presented at 250 pulses/s. Interaural level differences (ILD)  = 0 approximated levels which were most likely to be perceived as balanced. For normal hearing children, we measured behavioral thresholds to the click stimulus and presented stimuli in each ear at 40 dB above threshold (sensation level (SL). By contrast, it was not possible to assume a constant range of current between threshold and comfortable listening levels in cochlear implant users. We therefore used electrophysiological measures of brainstem activity evoked by each implant to determine approximately balanced current levels at the upper part of the dynamic range [32]. In each child, auditory brainstem responses were recorded from each device using previously reported stimulating and recording parameters (detailed in Table 2). Clear responses were recorded in all children at a range of current levels. Levels at which amplitudes were largest and most similar between the two devices were used as a first approximation of ILD = 0. All children were asked prior to testing whether the levels were “balanced” or “the same” between the implants. A perceived weighting of levels on one side was adjusted by reducing the level presented to that ear/by that device until the child perceived the bilateral levels to be “balanced” or “the same”.

**Table 2:** Auditory brainstem recording parameters.

Parameters	EABR (2-channel)
Stimulus parameters:	
Stimulus type	biphasic monopolar 1+2*
rate/sec (Hz)	11
N. of pulses/train	
rate/train	
Duration (ms)	
Pulse width (µs/phase)	25
Inter stimulus interval (ms)	91
Recording parameters:	
time window (ms):	
start	−5
end	80
Amplifier setting:	
low pass filter (Hz)	3000
High pass filter (Hz)	10
	
Artifact rejection:	
start (ms)	5
end (ms)	8
min (µs)	−30
max (µs)	30
	
Acquisition:	
A/D rate	2000
Baseline correction:	
start (ms)	−5
end (ms)	0

*monopolar 1+2 =  reference to CI plate and ball electrodes

Differences between levels were introduced while holding the overall current delivered constant; current increases in one device occurred with equal decreases in current in the other. The manufacture defined Clinical Units (CU) were used during testing because these are logarithmic values and provide linear increases/decreases (ILD = ±20, ±10, ±6, ±2 CU, where + is weighted to Left side/CI2 and – to Right side/CI1) but the conversion to standard current (amperes) varies slightly with device. Table 3 provides the details of all current levels used in each ILD condition for each group of bilateral cochlear implant users: A) the adolescent group and B) the more experienced bilateral users). Stimulus levels (dB SL) of acoustic clicks delivered to participants with normal hearing are indicated in Table 3C. Interaural timing differences (ITD) were presented at ILD = 0 (±2000, ±1000, ±400 µs, where + leads from Left side/CI2 and – leads from Right side/CI1). All but ITD = ±400 µs are beyond the normal physiological range of ITD perception but were included because children with bilateral implants had previously not been able to detect even these extreme differences [32]

**Table 3:** A) Stimulation current levels (dB re: 100 µA) in the group of adolescent cochlear implant users, B) stimulation current levels (dB re: 100 µA) in the experienced bilateral cochlear implant users implanted sequentially or simultaneously, C) stimulation levels in dB SL (Sensation Level, re: behavioral threshold) in the group with normal hearing.

			Devices N24					ILDs weighted to left/CI-2					ILD = 0					ILDs weighted to right/CI-1					dB difference
		n	K:M:RCS:RE:513		20		10		6		2		0		−2		−6		−10		−20		ILD = 0
	Duration of bilateral experience		Left	Right	Left	Right	Left	Right	Left	Right	Left	Right	Left	Right	Left	Right	Left	Right	Left	Right	Left	Right	
A)																							
Adolescents with long inter-implant delays*	0.14±0.01	34	0:3:1:13:17	1:8:15:5:5	8.16±1.16	7.76±1.21	7.91±1.12	8.33±1.11					7.66±1.17	8.33±1.11					7.42 ± 1.29	8.62 ± 1.19	7.16 ± 1.48	8.91 ± 1.35	0.76 ± 1.31
	0.86±0.03	29	0:3:1:12:13	1:7:11:5:5	8.00±1.22	7.99±1.14	7.86±1.20	8.25±1.15					7.72±1.20	8.52±1.26					7.58 ± 1.45	8.84 ± 1.36	7.44 ± 1.74	9.04 ± 1.67	0.80 ± 1.59
B)																							
Children with long inter-implant delays	5.30±1.55	16	0:1:6:9:0	0:2:8:6:0			6.37±1.36	8.01±1.61	6.54±1.36	7.84±1.60	6.70±1.37	7.67±1.60	6.78±1.37	7.59±1.60	6.87±1.37	7.51±1.60	7.03±1.37	7.34±1.60	7.19 ± 1.37	7.17 ± 1.60			0.80 ± 1.56
Children with no inter implant delays	4.31±1.10	29	0:0:0:28:1	0:0:0:28:1			7.11±1.12	8.15±1.22	7.26±1.12	7.99±1.22	7.42±1.12	7.84±1.22	7.50±1.12	7.76±1.21	7.56±1.12	7.68±1.21	7.73±1.12	7.52±1.21	7.89 ± 1.12	7.37 ± 1.21			0.26 ± 1.04
C)																							
Children with normal hearing	9.16±2.19	16	n/a	n/a			51.69±4.77	40.75±5.46	47.69±4.77	42.75±5.46	46.69±4.77	44.75±5.46	46.69±4.77	45.75±5.46	45.69±4.77	46.75±5.46	43.69±4.77	48.75±5.46	41.69 ± 4.77	50.75 ± 5.46			0.94 ± 3.04

*Data from newly implanted ears were combined in this group (i.e. ears were reversed for analysis for 6 adolescents with first CI on the left ear)

### Lateralization Task

All children were asked to indicate on which side of their head they heard the sound (left or right) by pointing or speaking. The first 23 of the 34 adolescents tested in the first months of implant use were provided with 2 additional choices (middle and both) as in a previous study [32]. Because they rarely gave these additional choices, the task was restricted to two choices (left and right) for all repeated measures after ∼10 months of bilateral implant use in this group as well as in the remaining 11 recruited adolescent participants and the other groups of children. Bilateral input across ILD and ITD conditions were randomly presented with presentations of unilateral stimuli. The first group of adolescent bilateral cochlear implant users listened to 2 conditions of unilateral stimuli (CI1, CI2) and 11 conditions of bilateral stimuli (+20 CU, +10 CU, −20 CU, −10 CU, −2000 µs, −1000 µs, −400 µs, +2000 µs, +1000 µs, +400 µs, 0 CU or µs difference). All 13 conditions were randomized and presented 6 times each. The remaining participants also listened to 2 conditions of unilateral stimuli (CI1/right, CI2/left) and 11 conditions of bilateral stimuli but the range of binaural differences was slightly narrower and there was an additional ILD condition and one less ITD condition (+10 CU, +6 CU, +2 CU, −10 CU, −6 CU, −2 CU, −1000 µs, −400 µs, +1000 µs, +400 µs, 0 CU or µs difference). All 13 conditions were randomized and presented 10 times each.

### Analyses

Repeated measures ANOVAs were used to assess change in response proportions with ILD and ITD conditions. Binary logit regression was used to fit responses across ILD and ITD conditions in each participant. ILD and ITD slopes were calculated using a bias-reduction general linear model; a t-test was used to compare slopes between bilateral groups. Linear regression was used to assess time-intensity trading by fitting predicted ITD for a centered perception against ILDs at predicted balance. Two way repeated measures ANOVAs were used to test for effects of ITD, group and ITD*group interactions. Significance was considered at p<0.05. Bonferroni and Dunnett T3 adjustments were used for post-hoc ANOVA comparisons.

## Results

### Perception of binaural level cues is rapidly established in most children with bilateral cochlear implants

**Figure 1:**
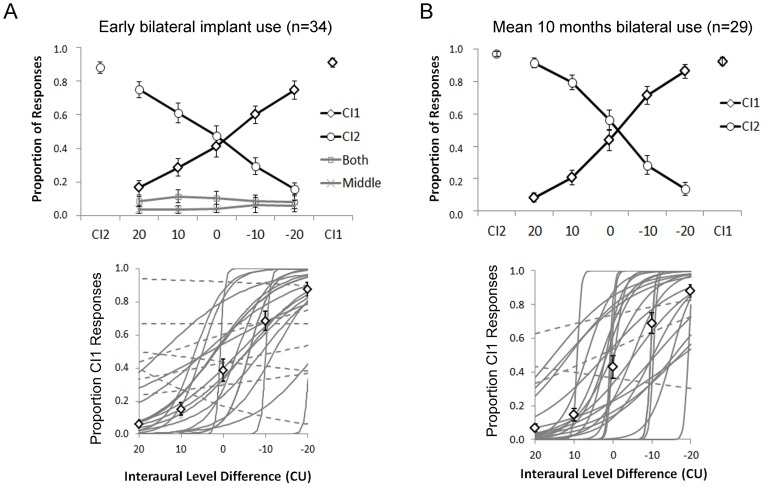
A)Thirty four adolescents responded to bilateral input either with no intended interaural level differences (ILD = 0) as indicated by equal amplitudes of auditory brainstem responses or with ILDs of 10 or 20 weighted to the second CI (positive values) or first CI (negative values) at early stages of bilateral implant use (0.14±0.01 year). Unilateral stimuli (CI1 and CI2) were also presented randomly to ensure the task was understood. Upper plot: Mean (±1 SE) proportion of responses from all participants. Mean responses to unilateral stimuli were>0.88 accurate. Significant changes in mean proportion of responses occurred with changing ILD (CI1 responses: p<0.0001, CI2 responses: p<0.001). ILDs of 20 CU (+ values indicate CI2 weighted and – values indicate CI1 weighted) were perceived to come from the correct side in 0.75 of trials with decreasing certainty for ILDs of 10 CU ILD and no significant difference between proportion of CI1 and CI2 responses when no ILDs were provided (ILD = 0) (p>0.05). Lower plot: Logit regression analyses of the proportion of right responses from each child revealed significant changes in 21 participants (solid lines) and non-significant changes in 9 participants (dashed lines). Data from 4 children was excluded from analysis because <0.67 of responses to unilaterally presented stimuli were accurate. White diamonds represent mean (±1 SE) data from significant regression curves. B) ILD perception was retested after 0.86±0.03 year of bilateral use in 29 of the 34 children. The “middle” and “both” choices were not accepted at this test time. Upper plot: significant change in mean responses were found with changing ILD (CI1 and CI2 p<0.0001). Lower plot: 25 of 29 adolescents showed significant changes in proportion of CI1 responses with ILD (solid lines), 3 had insignificant changes in responses (dashed lines), and data from 1 participant was excluded because responses to unilaterally presented stimuli were accurate on <0.67 of trials. White diamonds represent mean (±1 SE) data from significant regression curves.

Although children using bilateral cochlear implants were previously found to detect changes in inter-implant current levels (measured in dB re: 100 µA), it is not clear whether these skills required bilateral implant experience to develop nor whether this ability would be present in adolescents who, by virtue of their age and unilateral CI use, were questionable candidates for bilateral implantation [41]. To answer these questions, we assessed the ability of 34 adolescents with bilateral cochlear implants to detect differences between the intensity levels of current stimulation provided to their two devices. These study participants received a single cochlear implant (28 R: 6 L) at young ages (3.62±2.24 years old) and used it to develop hearing and spoken language for many years (9.02±2.80 years) before receiving a second implant in their other ear at 12.64±3.49 years of age. In Fig. 1A, the mean (SE) perceived locations of the bilateral stimuli within the first months (1.66±0.80 months) of bilateral implant activation are shown for bilateral stimuli which increased from higher weighted current in the second implant (positive ILD values) to equally weighted current (ILD = 0) to current weighted to the first implant (negative ILD values). Using the same behavioral task, we previously showed that children with normal hearing indicate similar acoustic stimuli are heard as coming from the side of the head as the more heavily weighted level of input (dB SPL) [32]. Equally weighted bilateral stimuli are heard as coming from the middle of the head. By contrast, a subset of the present cohort of adolescent cochlear implants users (n = 23) rarely indicated that they heard bilateral stimuli as coming from the middle of their head across these ILD conditions (proportion of “middle” responses  = 0.05, 95%CI:−0.01 to 0.11). As shown by the mean data in Fig. 1A, when this subgroup did indicate middle responses, they were no more prevalent for balanced bilateral stimuli (ILD = 0) than weighted stimuli (ILD>0>ILD) (F(4,19) = 1.0, p = 0.43). At ILD = 0, the proportion of responses to the second CI was 0.47±0.34 which was not significantly different from the proportion of responses to the first CI (0.41±0.34) (t(33) = 0.56, p = 0.58). We thus conclude that input at ILD = 0 was perceived as balanced. As bilateral input became increasing weighted to the first implant, the proportion of responses to that side increased (F(2,32) = 15.2, p<0.0001). Similarly, bilateral input became weighted in level to the second implant, responses to the side of CI2 increased (F(2,32) = 17.11, p<0.001). In the 23 children who were given 4 response choices, bilateral input was reported as coming from both ears occasionally (proportion of “both” responses  = 0.10, 95%CI: 0.04 to 0.15) with no effect of ILD condition (F(4,19) = 0.35, p = 0.84).

Because most responses were to the right or left side of the head, binary logit regression analyses were used to assess whether the increase in responses to the side of first implant increased as ILD became increasingly weighted to that side for each child as shown in Fig. 1B. Of the total 34 participants, data was excluded from 4 because the responses to unilateral stimulation were <0.67 accurate. In the remaining 30 children, 21 showed significant changes in their responses as shown by the thin grey lines corresponding with changes in ILDs at the first day of bilateral implant use and 9 had non-significant changes. Regression curves of non-significant changes with ILD (n = 9) are shown by the dashed lines. Mean ± SE data predicted by the 22 significant regression curves are shown by the diamond symbols. Detection of ILDs was measured again in 29 of these children after 10.08±1.55 months of bilateral implant experience. At this time, adolescents were asked only to indicate on which side of their head (right or left) they heard the sound. As shown in Fig. 1C, these participants showed balanced perception of input presented as ILD = 0 (the proportion of responses to first or second implant were not significantly different from one another (first CI: 0.44±0.34; second CI: 0.56±0.34; (t(27) = 0.97, p = 0.34) with increased proportion of responses to the side of weighted input (CI1: F(4,21) = 41.68, p<0.0001; CI2: F(4,21) = 41.75, p<0.0001). Binary logit regression curves fit to the proportion of responses to the first implanted side with ILD are shown in Fig. 1D; 25 of 29 are significant (solid grey curves), 3 were insignificant (dashed lines) and 1 was not considered for analysis because the child did not meet the task control criteria (≤0.67 proportion accuracy to unilaterally presented stimuli). Mean ± SE data predicted by the significant regression curves are shown by the diamond symbols. Overall, the data confirm ILD perception in most adolescents despite long periods of unilateral implant use prior to bilateral implantation and the very short period of bilateral implant experience.

### Sensitive perception of bilateral current level cues is present regardless of delay between implants

**Figure 2:**
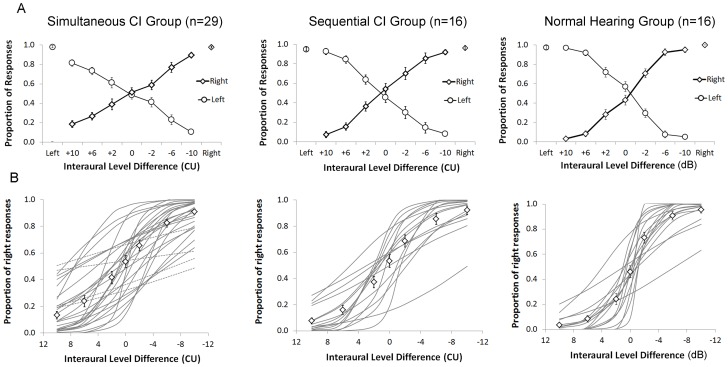
A) Mean (±1 SE) proportion of responses from all participants in three groups. Positive interaural level differences (ILDs) are left weighted and negative values are right weighted. CI groups had long term bilateral implant experience. Significant effects of ILD were found across groups (p<0.0001) with no effect of group (p>0.05) and no interaction (p>0.05). B) Logit regression curves for proportion of right responses from each participant for each group. Significant changes were found for all participants (solid lines) bar 4 of 29 children in the simultaneous group (dashed lines). Slopes were significantly reduced in the implanted groups relative to the Normal Hearing Group (p<0.0001).

**Figure 3:**
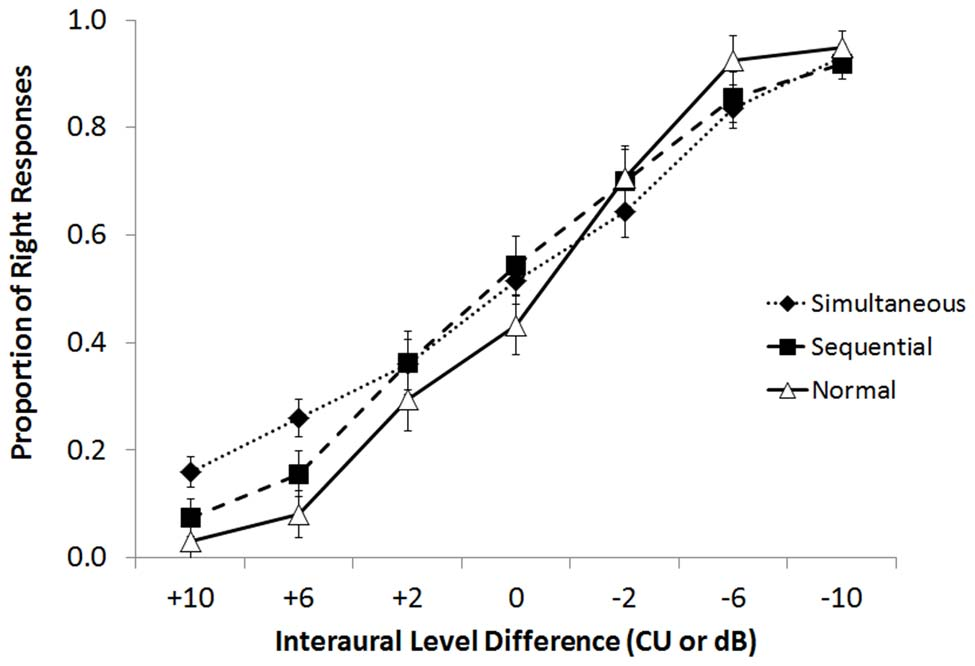
Experienced bilateral CI users and normal hearing peers perceived changes in ILDs as these cues moved from left to right weighted. Interaural level differences (ILDs) in CI users represent differences in CU and dB re: 20 Pa in the normal hearing group (∼0.08 dB re: 100 µA per acoustic dB change in ILD, see Table 3 for additional details). Significant effects of ILD were found across groups (p<0.0001). Rate of change in ILDs delivered by CIs (CU) was reduced relative to acoustic ILD (dB) (p<0.05) with no significant difference between the 2 implanted groups (p>0.05).

Given that perception of binaural level cues appeared so early in bilateral implant use and even in children who had very long delays between implantations, we asked whether there was an effect of the timing of implantation on this perception. In this part of the study, we measured proportion of responses to the left and right sides of the head in response to bilateral input in 3 groups of children matched for bilateral hearing age: 1) 29 children receiving bilateral implants simultaneously (unilateral implant use  = 0 years, bilateral implant use mean ± SD  = 4.31±1.10 years); 2) 16 children receiving bilateral implants sequentially (unilateral implant use  = 4.10±2.17 years, bilateral implant use  = 5.30±1.55 years); and 3) 16 children with normal hearing (age/bilateral hearing age  = 9.16±2.19 years). Mean (SE) responses in the 3 groups are plotted in Fig. 2A. Responses at ILD = 0 are not significantly different from the expected 0.50 in any group (Simultaneous: t(28) = 0.39, p = 0.70; Sequential: t(15) = 0.84, p = 0.42; Normal hearing: t(15) = 1.36, p = 0.20) indicating bilateral input in this condition was perceived as balanced for level. Proportion of responses to either side increased as ILDs weighted to that side increased. Binary logit regression curves fit to responses to the right/first implanted side are shown in Fig. 2B and revealed significant detection of ILDs in all (thin grey lines) but 4 children tested (dashed lines). All 4 of the children with non-significant changes were in the simultaneous group but were of similar ages (6.99±1.65 years) and had similar durations of bilateral implant use (4.08±0.92 years) as the other children in this group. Note that the ILD on x-axis is in CU for CI groups and dB re: sound pressure (20 µPa) for the normal hearing group. Table 3 indicates the current provided to each device/ear at each ILD for each group. Each change in ILD of acoustic stimuli by 1 dB is compared to ∼0.08 dB re: 100 µA of ILD change in CI stimulation. Diamond symbols in Fig. 2B denote mean (SE) data predicted by significant regression curves. Data from those children who showed significant changes in response with ILD are compared by group in Fig. 3. Repeated measures ANOVA testing revealed a significant effect of ILD on proportion of right/first implant responses as expected (F(6,49) = 189.8, p<0.0001), with no significant differences in responses between the groups (F(2,54) = 0.52, p = 0.60) or interaction between ILD and group (F(12,100) = 1.14, p = 0.34). The rate of ILD change was further calculated using a bias reduced general linear model. Comparisons by group with ANOVA showed a significant effect of group on the rate of change in proportion of right responses with ILD (F(2,54) = 7.07, p = 0.002). Post-hoc comparisons (Bonferroni) indicated that the rate of change in ILDs delivered by CIs was reduced relative to ILDs delivered by acoustic input (Simultaneous vs Normal: p = 0.002; Sequential vs Normal: p = 0.02) but that there was no difference between the CI groups regardless of prior unilateral implant exposure (p = 1.00).

### Perception of binaural timing cues is not present during the first year of bilateral implant use in children with long delays between implants

**Figure 4:**
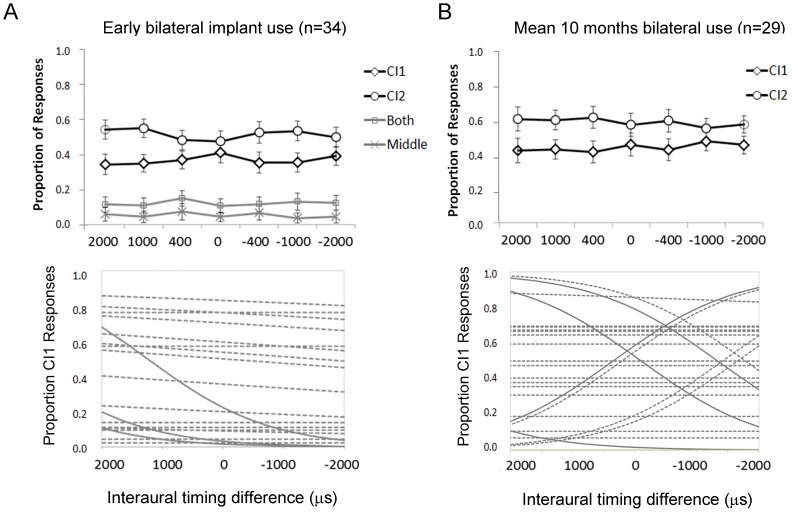
A) Upper plot: Mean (±1 SE) proportion of responses to changes in interaural timing differences (ITDs) at early stages of bilateral implant use (0.14±0.01 year). Positive values lead from the newly implanted ear (CI2) and negative values from the more experienced ear (CI1). Most responses are to either CI1 or CI2. No significant changes were found for CI1 or CI2 responses with ITD changes (p<0.05). Lower plot: Logit regression curves for proportion of right responses from each participant reveal non-significant slopes in 15 participants (dashed lines) and significant slopes (solid lines) in 4 participants but in the opposite direction from expected. Data could not be analyzed in 2 children as all responses were to CI2, 3 children were excluded from individual because of non-significant changes in responses to interaural level differences (shown in Figure 1A), and 1 was excluded because responses to unilateral stimuli were <67% accurate. B). Upper plot: No significant changes in CI1 or CI2 responses were found after 0.86±0.03 year of bilateral use. Lower plot: Logit regression curves showed non-significant changes in 17 participants (dashed lines) and significant changes in 6 participants (solid lines) although 1 was in the opposite direction from expected. Data could not be analyzed in 2 adolescents who indicated CI2 for all responses, 3 participants with non-significant changes with interaural level differences (Figure 1B) and 1 whose responses to unilaterally presented stimuli were <67% accurate.

In contrast to the encouraging findings with respect to detection of binaural level cues, the perception of interaural timing differences was not evident in the cohort of adolescents studied during the first year of bilateral implant use. Mean ± SE proportion of responses in the group tested after 1.66±0.80 months of bilateral implant experience are shown in Fig. 4A. As in the ILD conditions, the 23 adolescents who were given 4 response choices did not often choose either middle or bilateral responses and these did not change with ITD condition (Middle: 0.04±0.03(SE), F(4,19) = 1.0. p = 0.43; Bilateral: 0.12±0.03(SE), F(4,19) = 0.17, p = 0.95). As shown in Fig. 4A, the proportion of right responses in the total group was not affected by changes in bilateral timing from input which lead from the second implanted side (positive values) to input leading on the first implant side (negative values) (F(6.28) = 0.71, p = 0.64). To confirm this, binary logit regression was used to fit the proportion of right responses to the changes in ITDs for 19 of the 21 participants who had significant ILD perception (regression analyses could not be completed for the remaining 2 participants as all responses to ITDs were to the side of CI2). The resulting fit lines revealed that only 4 of the 19 adolescents had significant changes in responses with ITDs (solid lines) and all 4 were in the opposite direction to normal (the 15 insignificant curves are shown by the dashed lines). In Fig. 4B, mean ± SE proportion of responses measured in the 29 participants who returned for testing are plotted. At this time, responses were limited to either right or left but the cohort showed no evidence of change in their responses with changing ITDs (F(6,21) = 1.19, p = 0.35). To confirm this, logit binary regression analyses were completed for data from 23 of the 29 adolescents (3 had insignificant perception of ILDs, 1 had a <0.67 rate of accurate responses to unilateral presentations, 2 only responded to the side of CI2 for all ITD presentations). As shown, significant curves (solid lines) were found for only 6 of the 23 adolescents. Of these significant curves, 1 was in the opposite direction from normal. The majority of the curves (17/23) were not significant. Overall then, ITD perception was not present in most of this group of adolescents (19/23) at early stages of bilateral implant use. This finding was consistent with our reports from children who received bilateral cochlear implants sequentially and who were tested using a similar lateralization technique after 2.2 ± 1.1 years of bilateral implant use [32].

### Detection of binaural timing cues requires many years of bilateral cochlear implant use to develop

**Figure 5:**
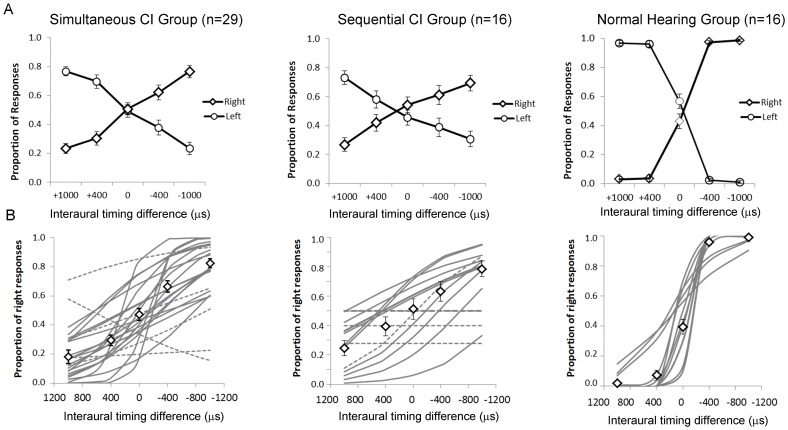
A) Mean (±1 SE) proportion of responses from all participants in three groups. Positive interaural timing differences (ITDs) indicate bilateral stimuli leading from the left and negative values indicate right leading stimuli. CI groups had long term bilateral implant experience (Simultaneous Group: 4.31±1.10 years; Sequential Group: 5.30±1.55 years). Mean responses from the Normal Hearing Group are essentially at ceiling for all ITDs>0. B) Logit regression analyses of data from individual children are shown (solid lines = significant regression curves; dashed lines = non-significant regression curves). Twenty of 25 in the Simultaneous Group significantly detected changes in ITD (data from 5 were not analyzed as detection of interaural level cues shown in Figure 2B was not significant). Eleven of 16 children in the Sequential Group showed significant changes. There was no significant difference between these proportions (Mann-Whitney U = 177.5, p = 0.42). Significant changes were found in all children in the Normal Hearing Group.

Based on the lack of ITD perception in the adolescent cohort, we asked whether these skills might develop in the best conditions, when bilateral cochlear implants are provided without delay in young children, and with long periods of bilateral implant use. Data in Fig. 5A demonstrate, for the first time, that children using bilateral cochlear implants show changes in mean (SE) proportion of responses to either side of their head with changes in ITDs. Bilateral stimuli leading from the second/left implant have a higher proportion of responses to that side and stimuli leading from the first/right implant have a higher proportion of responses to that side. This was true for both children who received their implants simultaneously and sequentially when they had had many years of bilateral hearing experience. Mean accuracy for children with normal hearing was very high for all stimuli leading from the left or right ears, revealing the ease of this task for this group. Responses from individual children were assessed using binary logit regression; significant curves (solid lines) and non-significant curves (dashed lines) are shown in Fig. 5B. Mean (SE) data predicted by significant regression curves are shown by the diamond symbols. All 16 of the children with normal hearing showed significant changes in responses to ITDs. Regression curves predict essentially perfect accuracy for 11/16 children with normal hearing (6.33–14.80 years of age) with excellent accuracy in 2/16 children (>90%, 8.47 and 11.03 years of age) and good accuracy in 3/16 children (>65%, 5.55 years, 7.80, and 7.95 years of age). Of the 25 simultaneously implanted with significant ILD perception, 20 showed significant changes in responses with ITDs (after 4.31±1.10 years of bilateral implant use) as did 11 of 16 children sequentially implanted after using their bilateral implants for 5.30±1.55 years. There was no significant difference between these proportions (Mann-Whitney U = 177.5, p = 0.42). It was not possible to account for which bilateral implant users acquired ITD perception based on duration of bilateral implant use (t = 1.2, p = 0.23), inter-implant delay (t = 1.0, p = 0.33), or age at first implant (t = 0.25, p = 0.80) (R = 0.20, F(3,44) = 0.55, p = 0.65).

### Perception of binaural timing cues is different from normal and affected by delay between implantations

As mentioned above, the proportion of right responses to bilateral input presented without level differences (ILD = 0) or timing differences (ITD = 0) were not significantly different than the proportion of left responses in children implanted bilaterally in simultaneous or sequential procedures. Nonetheless, as shown in Figs. 1 and 2, binary logit curve analysis provided a range of ILDs and ITDs at which the proportion of responses to the right/first implant side would be predicted to be 0.50, or balanced, for each child. ILDs predicted at negative values for 0.50 response rates mean that responses measured at ILD = 0 were weighted toward the opposite (left or second implant) side. By the same token, the right/first implant is weighted at ILD = 0 when positive value predictions of 0.50 responses rates occurred. We asked whether these ILD shifts corresponded to compensatory shifts of ITDs in the opposite directions.

**Figure 6:**
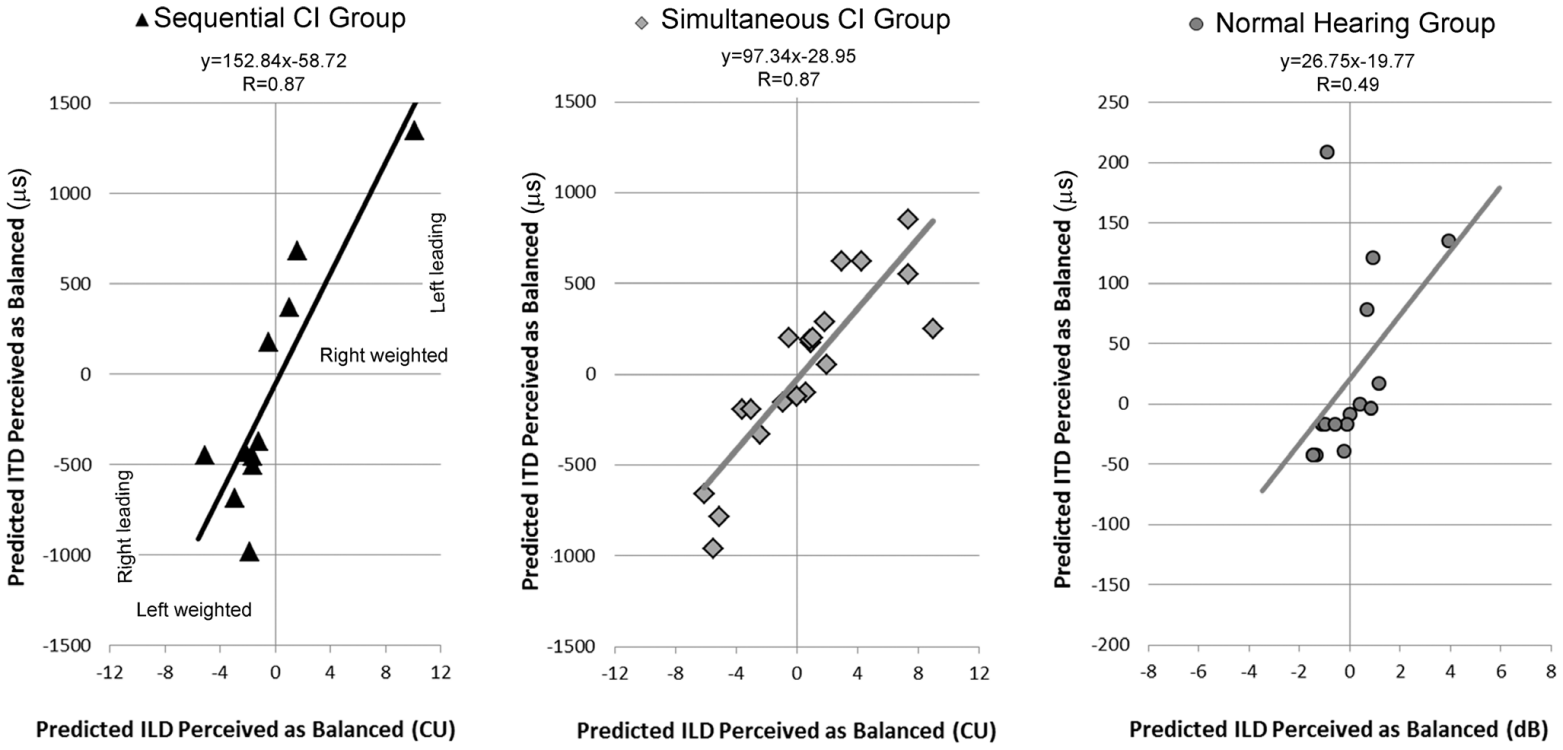
Logit regression curve analyses from **Figures 3B** and **5B** were used to calculate predicted balance (0.5 proportion of right responses) for both interaural level and timing differences (ILDs and ITDs), revealing time-intensity trading in all groups. Predictions of negative ILDs for balanced bilateral input indicated that bilateral stimuli at ILD = 0 was left weighted and predictions of positive ILDs for balanced bilateral input indicated ILDs = 0 was right weighted. The perception of ITDs to stimuli presented at ILD = 0 shifted predictably in each group; linear regressions were significant in both CI groups (p<0.0001) with a trend in the Normal Hearing Group (p = 0.054). Left weighted stimuli shifted balanced perception toward right leading stimuli and right weighted stimuli shifted balanced perception toward left leading stimuli.

In Fig. 6, the predicted balanced ITD is plotted against the predicted balanced ILD for each of the simultaneously and sequentially implanted children. Negative ILD values indicate that predicted balance was weighted to the right implant, meaning that ITDs were actually presented with left weighted bilateral input. Positive ILD values indicate presentation of right weighted stimulation levels. Linear regression lines demonstrate a significant relationship between the current levels which were presented at ILD = 0 and ITDs leading in the opposite ear predicted to be balanced at the levels presented for the CI groups (Sequential: R = 0.88, p<0.0001; Simultaneous: R = 0.87, p<0.0001) and a trend for the Normal Hearing Group (R = 0.49, p = 0.054). The sequential group showed a compensatory shift of (mean ± SE) 152.84±28.30 µs for every 1 CU (0.08 dB) that bilateral levels were presented off the predicted balance (95% confidence interval: 88.83 to 216.84 µs). The simultaneous group showed a slightly smaller compensatory shift of 97.34±13.06 µs for every 1 CU (0.08 dB) that bilateral levels were presented off the predicted balance (95% confidence interval: 69.90 to 124.78). The difference between groups was not significant (overlap between 95% confidence intervals for these slopes). Despite similar perception of ILDs when dB current changes were compared with dB acoustic changes, the normal hearing group showed compensatory shifts of 26.75±12.72 µs with every 1 dB bilateral clicks were presented off balance (95% confidence interval: −0.53 to 54.02) as plotted in Fig. 6. These confidence intervals were narrower than either bilaterally implanted group reflecting the significant abnormality in how implanted children used binaural timing cues to compensate for small shifts away from balanced implant levels.

**Figure 7:**
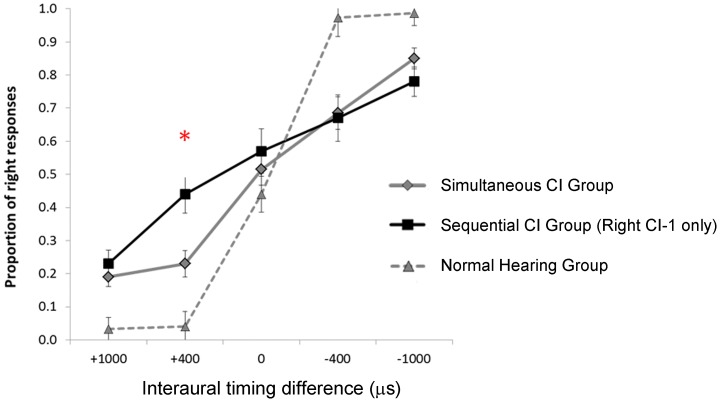
Mean (±1 SE) proportion of right responses from children with significant changes in interaural timing differences (ITDs) from the Normal Hearing Group (n = 16), the Sequential CI Group (10 with CI1 in the right ear), and the Simultaneous CI Group (n = 20). Responses changed significantly with ITD (p<0.0001) with no group effect (p>0.05) but with a group*ITD interaction (p<0.0001). The proportion of right responses for stimuli left leading by 400 µs was significantly greater in the Sequential CI group (p<0.005). The rate of change was significantly reduced in the CI groups relative to normal (p<0.0001) and there was a trend toward reduced rates of change in the Sequential CI group relative to the Simultaneous CI group (p = 0.05).

Additional group differences are shown in the mean (SE) proportion of right responses with changes in ITD plotted in Fig. 7. Only children who showed significant detection of ITDs based on binary logit regression analyses (Fig. 5) were included in Fig. 7 and corresponding analyses. Data from 1 child in the sequential group with significant ITD detection were excluded because this was the only child who met the criteria for analysis to have been implanted in the left ear first. Because ITDs were presented at the levels in the ILD = 0 (balanced) condition, responses at ITD = 0 (no timing differences) were not significantly different from chance (0.50) in any group (Simultaneous: t(19) = 0.31, p = 0.76; Sequential: t(9) = 1.05, p = 0.32; Normal: t(15) = −1.36, p = 0.19). Overall, right responses were less prevalent when bilateral input lead in the left or second implant (positive ITDs) and more prevalent when bilateral input lead in the right or first implant (negative ITDs). Repeated measures ANOVA revealed a significant change in proportion of right responses with changing ITD (F(4,40) = 172.66, p<0.0001) but no overall significant differences between groups (F(2,43) = 0.47, p = 0.67). A significant ITD*group interaction (F(8,82) = 7.02, p<0.0001) was found; children using bilateral implants were less certain of their responses to ITDs than their peers with normal hearing for all ITDs> 0 (p<0.005). Of particular interest, in the +400 µs condition (bilateral input leading in the second implanted or left ear), the Sequential Group showed a significantly larger proportion of responses away from this side and toward to their first implanted right side than the Simultaneous group (p<0.005). This bias was no longer present when a longer ITD leading from the left (1000 µs) was presented (p = 0.43). No significant differences between the proportion of responses between the bilaterally implanted groups were observed for right/first implant leading ITDs (−400 µs: p = 0.86; −1000 µs: p = 0.20). This indicates a weighted perception of binaural timing cues toward the ear first implanted in the Sequential group for ITDs leading away from that side by 400 µs. A more specific analysis of the rate of change in right responses with ITD for each child was achieved using a biased reduction general linear model. Group effects were confirmed by ANOVA (F(2, 43) = 39.62, p<0.0001). Post-hoc comparisons (Dunnett T3) revealed reduced rates of change in the simultaneously and sequentially implanted groups relative to the Normal Hearing Group (p<0.0001 and p<0.0001, respectively) and a trend toward reduced change in the Sequential group compared to the Simultaneous group (p = 0.05). Reduced slopes were associated with longer inter-implant delays (R = −0.37, p = 0.04) and shortened hearing experience (R = 0.42, p = 0.003) but not age at test (R = 0.11, p = 0.47), age at first implant (R = 0.25, p = 0.17), age at second implant (R = −0.08, p = 0.67) or duration of bilateral implant use (R = −0.19, R = 0.30).

## Discussion

In the present set of experiments, we took an optimistic view and hypothesized that perception of binaural cues could be established in children who are deaf in both ears. In support, detection of interaural level differences was found to be present at very early stages of bilateral implant use even in adolescents who had developed hearing from an implant in one ear for most of their lives. Moreover, children with longer term bilateral implant use responded to changes in interaural level differences similarly to their normal hearing peers with no apparent effects of unilateral hearing before bilateral implant use. Even more encouraging, we provide the first evidence that these children were also able to detect interaural timing cues. These skills took many years to develop as they were not evident in the present cohort of adolescents who recently received bilateral implants nor in a younger group of children tested after 2.2±1.1 years of bilateral implant use as previously reported [32]. Along with the extended period required for detection of binaural timing to develop, children with bilateral implants were less certain in their responses to binaural timing cues than their normal hearing peers and perception of binaural timing cues was affected by an inter-implant delay. Children implanted sequentially showed an increased sensitivity to ILD cues than ITD cues compared with children implanted simultaneously and increased responses to their first implanted side for sounds leading in the opposite ear by 400 µs. These differences are consistent with functional asymmetries previously reported in the central auditory pathways in children who receive bilateral implants after>1.5 years of unilateral implant use [36], [37].

### Establishing binaural level perception in children and adolescents who are deaf

Data presented in Fig. 1 demonstrate that establishment of binaural level perception occurs rapidly after bilateral implantation in adolescents despite the very short term exposure to stimulation in the newly implanted ear relative to its much longer duration of deafness. Thus, the basic mechanisms for binaural processing of level cues appear to be retained in these children and can be evoked by electrical stimulation using a pair of electrodes in the apical ends of the two implant arrays. Electrophysiological evidence of binaural interaction at the level of the brainstem in children receiving bilateral implants is consistent with this suggestion [36]. The Binaural Difference, the calculated difference between brainstem responses to bilateral input and the sum of both unilaterally evoked responses, decreased in amplitude with increases in ILD. In addition, as the Binaural Difference amplitude decreased, perception of the bilateral input increased on one side of the head. The participants in that study had longer bilateral implant experience and were younger at the time of testing than the adolescents represented in Fig. 2; nonetheless, it is reasonable to suggest that the pathways required for binaural processing of level cues are functional in the present cohort of adolescents despite the long term unilateral stimulation of their right ear and deprivation of their left ear. This is remarkable considering persistent asymmetries along the developing bilateral auditory pathways after unilateral implant use/deprivation, including faster brainstem activity from the first stimulated ear [36] and abnormally strong responses in auditory cortices promoted from the first implanted ear [37].

Further evidence that bilateral and unilateral deprivation in development did not eliminate ILD perception is shown in Fig. 2. These data, from children with longer term bilateral implant experience, reveal that detection of ILDs was not significantly different between children who had received bilateral implants after a period of unilateral implant use and children who received both devices simultaneously. This occurred despite the fact that the sequentially implanted group often used 2 different cochlear implant devices. The perceived changes in response to ILDs shown in Fig. 3 indicate that children using bilateral implants have similar sensitivity to ILDs as those reported in adult bilateral implant users [19], [20], [25], [28] albeit significantly reduced relative to that in normal hearing children (when acoustic hearing is replaced by current). Thus, these data demonstrate that ILD processing is retained in the brainstem despite bilateral deafness in childhood or unilaterally promoted changes to the auditory pathways and, moreover, that binaural coding of level cues is established early in development and is not easily lost. This is consistent with the suggestion that the mechanisms for ILD coding are so integral to survival that they have been conserved for almost 200 million years [1]. Remarkably, the same mechanisms can be evoked by a range of electrical current provided by cochlear implants in children.

The rapid ability by children who are deaf to detect binaural level cues is clinically important because cochlear implant levels must be customized for each child. The behavioral lateralization task used in the present study could help to establish bilaterally balanced perception even in early stages of device use. Although electrophysiological measures can be used to help approximate balanced bilateral levels, this must be confirmed behaviorally [32], [42]. As shown in Fig. 6, if ILDs are not properly calibrated/balanced, large offsets in ITDs occur (∼150 µs/dB on average in sequentially implanted children and ∼95 µs/dB in simultaneously implanted). Increased vigilance about providing balanced bilateral levels could give children access to more accurate ITD cues and thus facilitate development of ITD perception. The current practice of setting current levels in each implant independently, with measures that use step sizes of 5 CU (∼7–9.5 dB current), might be allowing offsets in binaural timing perception of many hundreds of µs to be present, particularly in sequentially implanted children. This could be avoided by exploiting the ability of children and adolescents to detect small differences in intensity between their two implants.

### Development of binaural timing perception in children and adolescents who are deaf

Unlike the rapid restoration of binaural level perception, adolescents receiving bilateral cochlear implants were unable to detect even very large differences in binaural timing during the first year of bilateral implant use as shown in Fig. 4. This is consistent with our previous work which revealed no significant detection of ITDs by children who had 2.2±1.1 years of bilateral implant experience [32]. Similarly, Litovsky and colleagues [18] reported no clear ITD perception in a group of bilaterally implanted adults with pre-lingual deafness as compared with adults whose deafness was acquired later in life (i.e. after binaural hearing had developed).

The first evidence to our knowledge that children who are deaf can develop ITD perception through long term bilateral cochlear implant use is shown in Fig. 5; both children implanted simultaneously and sequentially significantly perceived changes in ITDs. A high degree of variability was found in both groups, consistent with results in adult bilateral users; while some adults can hear differences between the ears of as little as ∼100 µs others require much longer ITDs [18], [20]. Although it is clear that the bilateral electrical input is integrated in the auditory brainstem of children who are deaf [36], it is not clear whether normal processing is occurring. ITD coding in humans is thought to involve coincidence detectors [43] in the medial superior olive; the time delay is accounted for changing axonal properties and/or synaptic inhibition [44], [45]. Both hemispheres of the bilateral pathways work in concert with increased inhibition in pathways ipsilateral to ear with the leading input (closest sound source) [1]. It is unclear whether these processes are occurring in children using bilateral implants. Inhibitory inputs are disrupted by abnormal binaural input during development [46]–[48] and asymmetries between the cortical hemispheres persist after unilateral cochlear implant use [37], leading to an “aural preference” for the hearing ear [49]. Moreover, the children with bilateral implants may not be hearing the ITD changes in a normally expected way; for example, they may not perceive a fused image moving from one side of the head to the other. Binaural fusion is not required for ITD detection [22] but it may reduce efficient processing of the bilateral input, compromising its use in restoring meaningful and useable binaural hearing.

### Persistent abnormalities in binaural timing perception in children using bilateral implants

Although children using bilateral implants develop perception of binaural cues after long term bilateral implant use, differences from normal remain. As shown in Fig. 6, children make adjustments in perception of timing cues to compensate for slightly off balanced bilateral input levels. Linear regression analyses suggest that for every 1 dB shift, children with normal hearing compensate by using ∼27 µs leading in the opposite ear to restore bilateral balance, consistent with the normal ITD sensitivity range of 15–30 µs [50]. Children with bilateral implants, by contrast, show remarkably large reliance on binaural level cues relative to timing cues as very small shifts in ILD away from balance required very large adjustments in ITD toward the opposite ear (∼95 µs in the simultaneous group and ∼150 µs in the sequential group). This likely reflects abnormally poor sensitivity for ITDs which is supported by the data presented in Fig. 7. Although children using bilateral CIs successfully detect changes in ITDs, they do so with less accuracy than children with normal hearing. Responses from children with normal hearing were near ceiling (proportion of right responses = 0.0 or 1.0) for all ITDs other than ITD = 0, indicating that the task was very simple for them. The reduced accuracy in children using bilateral cochlear implants in response to these very large ITDs highlights their profound impairment to perceive ITDs within the first years of bilateral use ([32] and Fig. 4) and reiterates the poor ITD sensitivity which is restored after much longer term bilateral use (Fig. 5).

It is not entirely surprising that children with bilateral cochlear implants acquire abnormal ITD sensitivity. Their abilities were in line with that shown in adult bilateral implant users who had normal hearing in both ears before the onset of deafness [20]. This suggests abnormalities in ITD perception are affected significantly by the limits of cochlear implant stimulation. Nonetheless, altered development also plays a role in ITD perception in these children as shown by the increased proportion of responses to the first implanted ear in children sequentially implanted when stimuli leads from the other side and a trend toward reduced change in perception of right responses with ITD compared to children simultaneously implanted. The apparent preference of the first implanted ear is consistent with findings of persistent asymmetry in the auditory pathways in children who developed hearing though unilateral cochlear implants for longer than 1.5 years [36], [37] and the “aural preference syndrome” which occurs with unilateral deprivation during early auditory development [49].

We have shown in the present study that ITD detection in children using cochlear implants is an emerging skill which leads us to ask whether these skills might further develop with time. It is true that the children with normal hearing had longer binaural hearing on average than the bilateral cochlear implant users (9.16 ± 2.19 versus 5.30 ± 1.55 years in the sequential group). Yet, even the youngest children with normal hearing, aged 5.55 years and 6.33 years, detected the ITD cues presented highly accurately and it is not clear whether children with bilateral cochlear implants, even those receiving bilateral implants simultaneously, would outperform adult CI users. Methods which seek to better match the place and level of stimulation between the devices, as discussed above, could help both adults and children using CIs along with better coordination of temporal cues between the devices to help ensure binaural fusion of the input. In addition, behavioral therapy might help bilateral implant users increase their awareness of these important binaural cues and translate them into useable improvements in sound localization and speech recognition in noise.

### Summary and Conclusions

Despite bilateral deafness in early childhood, interaural level cues were available almost immediately to most bilateral implant users. The emerging detection of timing cues after many years of bilateral cochlear implant stimulation reflects the plasticity of the developing system whilst the findings of preferred responses toward a unilaterally stimulated ear during development demonstrate that limitations to auditory development remain. Nonetheless, the restoration of binaural cues through bilateral cochlear implantation is a marked endorsement of this clinical intervention and highlights the potential for children to recover, at least to some extent, a missing sensory ability.
